# A new era for Advances in Laboratory Medicine

**DOI:** 10.1515/almed-2023-0116

**Published:** 2023-09-13

**Authors:** Álvaro González, Roser Ferrer-Costa, Pilar Fernández-Calle, Ignacio Arribas, Wladimiro Jiménez

**Affiliations:** Clínica Universidad de Navarra, Pamplona, Navarra, Spain; Service of Biochemistry, Vall d’Hebron University Hospital, Barcelona, Spain; Service of Clinical Biochemistry, La Paz University Hospital, Madrid, Spain; Service of Clinical Biochemistry, Ramón y Cajal University Hospital, Madrid, Spain; Service of Biochemistry and Molecular Genetics, Hospital Clinic Universitari de Barcelona, Barcelona, Spain

**Keywords:** PubMed, Scopus, Journal Citation Reports, impact factor, SCImago

The Editorial of the first issue of Adv. Lab. Med. included a description of the goals of this new scientific journal, which comprised achieving a significant presence in impact factor indices [1]. The first step to achieve this goal was to get this journal indexed in the search engines of the most relevant biomedical databases. Otherwise, dissemination would be restricted to a relatively small community of readers. Nowadays, our journal is available on the most important biomedical databases, including PubMed Central (National Center for Biotechnology Information, NCBI); Web of Science (Clarivate Analytics); and Scopus (Elsevier). Hence, search engines provide open access to the works published in our journal to the whole scientific community, which enhances the visibility of our articles. However, it is worth mentioning that, before our journal was included in these search engines, it already received a high number of visits and citations, what demonstrates the quality of and interest raised by our articles. Our journal publishes original articles, systematic reviews and also scientific recommendations submitted by the different Working Groups of the Spanish Society of Laboratory Medicine.

The quality and prestige of a scientific journal is measured by a range of quantitative bibliometric indices, which also suggest the difficulty in getting an article published in that journal. The most widely used bibliometric indicator is impact factor (IF), published by the Journal Citation Reports (Clarivate Analytics) on the basis of information available on Web of Science. IF is calculated as the average number of citations of items published in the journal during the preceding two years [2]. Thus, the 2022 IF would correspond to the number of citations of articles published between 2021 and 2020 during 2022, divided by the number of articles published in 2021 and 2020. Another important bibliometric indicator is the position of a journal within its group. In the case of Adv. Lab. Med., this group includes Medical Laboratory Technology, which embraces the most relevant journals in the field of clinical biochemistry, such as Clinical Chemistry or Clinical Chemistry and Laboratory Medicine. Other quality indicators include SCImago Rank (Elsevier), based on the information contained in the Scopus database. The SCImago Journal Rank (SJR) is the alternative quality indicator to IF. This rank is calculated using an algorithm that accounts for the mean number of citations received by a journal during the preceding 3 years during a year and the prestige of the journals where the citations come from. This year, our journal was indexed and ranked by these databases and others, such as Google Scholar Metrics.

Regardless of the initial position achieved on bibliometric rankings, fulfilling this goal in such a short period of time was a remarkable success. Of note, our journal was indexed by IF and SJR starting with the first year of publication, which indicates that our journal was rapidly included in bibliometric indices. This success is the result of the large amount of effort and enthusiasm put by our editorial team, added to the rigor and quality required to all authors and, especially, the trust that authors have placed on this project. Of special mention is the major role of reviewers, whose voluntary work is essential to maintaining our journal to the highest standards.

Fulfilling these goals will open new opportunities for the prestige or our journal to keep growing via the publication of good science. This will probably imply in the future adopting more strict requirements to ensure the general and methodological quality of the works published in our journal. The quality of the scientific production of a researcher is primarily measured by the number of publications in indexed journals. Therefore, the increased visibility of our journal, added to our IF, will encourage more and more authors to submit their works to us. We face a new era that will be even more stimulating than the first period of life of this journal.
